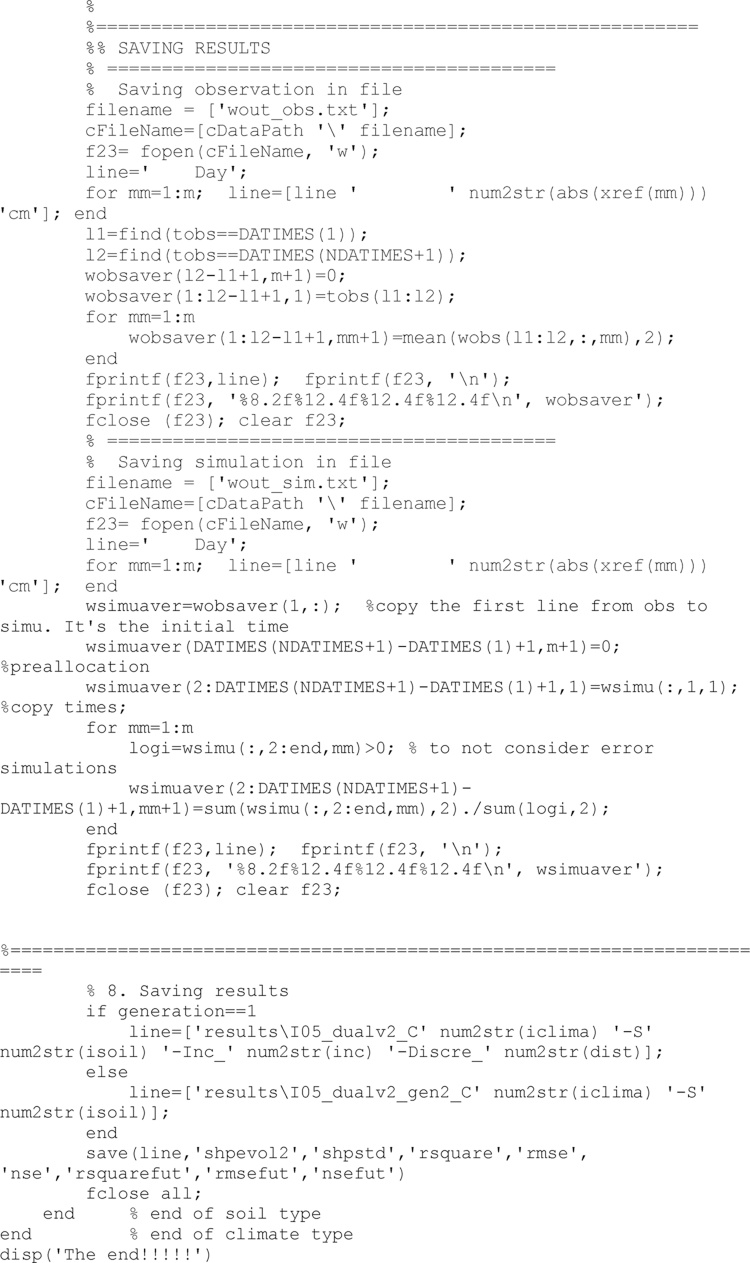# MATLAB algorithm to implement soil water data assimilation with the Ensemble Kalman Filter using HYDRUS

**DOI:** 10.1016/j.mex.2018.02.008

**Published:** 2018-03-07

**Authors:** Javier Valdes-Abellan, Yakov Pachepsky, Gonzalo Martinez

**Affiliations:** a Department of Civil Engineering, University of Alicante, Alicante, Spain; b USDA-ARS, Environmental Microbial and Food Safety Lab., Beltsville, MD, USA; c Department of Applied Physics, University of Córdoba, Córdoba, Spain

**Keywords:** Climate/soil EnKF efficiency, Hydrus, EnKF, Soil water flux modelling

## Abstract

Data assimilation is becoming a promising technique in hydrologic modelling to update not only model states but also to infer model parameters, specifically to infer soil hydraulic properties in Richard-equation-based soil water models. The Ensemble Kalman Filter method is one of the most widely employed method among the different data assimilation alternatives. In this study the complete Matlab© code used to study soil data assimilation efficiency under different soil and climatic conditions is shown. The code shows the method how data assimilation through EnKF was implemented. Richards equation was solved by the used of Hydrus-1D software which was run from Matlab.

• MATLAB routines are released to be used/modified without restrictions for other researchers
• Data assimilation Ensemble Kalman Filter method code.
• Soil water Richard equation flow solved by Hydrus-1D.

## Graphical abstract

**Figure:**
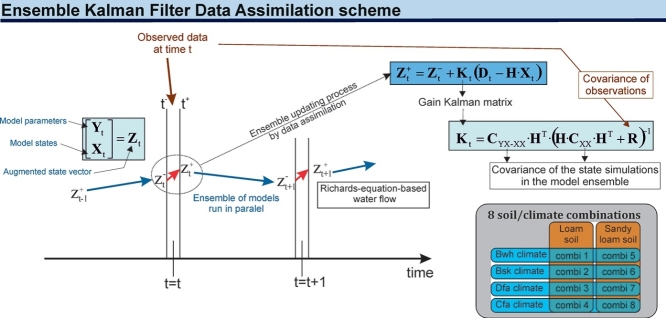

**Specifications Table**

**Table:** 

Subject area	*Select one of the following subject areas:*
	• *Agricultural and Biological Sciences* • *Computer Science* • *Engineering* • *Environmental Science*
More specific subject area	*Data assimilation by Ensemble Kalman Filter applied to soil water flux modelling to infer soil hydraulic properties.*
Method name	*Climate/soil EnKF efficiency.*
Name and reference of original method	
Resource availability	

## Method details

Data assimilation, DA, methods improve the model performance by integrating observed data (i.e., system states) into the modelling process in order to correct the model predictions and or model parameters [[1], [2]]. Among the different DA alternatives, the Ensemble Kalman Filter (EnKF) is one of the most widely used DA methods [[6], [8]]. Shortly, an ensemble of models is randomly generated, then propagated in time to the to the next update event. For each update event, a state error covariance matrix is calculated from the state values simulated by the different ensemble members before the update (a priori). A covariance matrix of observations is also obtained at the same time. Both covariance matrices are used to obtain a new set of model states and model parameters.

In the present contribution, we share the Matlab code used in Valdes-Abellan et al. [3] to apply the EnKF data assimilation method to soil water flow modelling. The code was employed to infer soil model parameters by updating both states and parameter according to the approach showed in Chen and Zhang [7].

The code was created considering a 1-layer soil profile; nevertheless, is straightforward modifying and adapting the code to more complex profiles. Additionally, it is prepared to consider different climates and soil types. This feature can also easily modify to be adapted to the new user aims.

All required subroutines and other files required to run the program are included in the present study as Supplementary materials.

## Procedure

This lines are not required to run the model but to clearly exposed the variables used in the model

**Figure:**
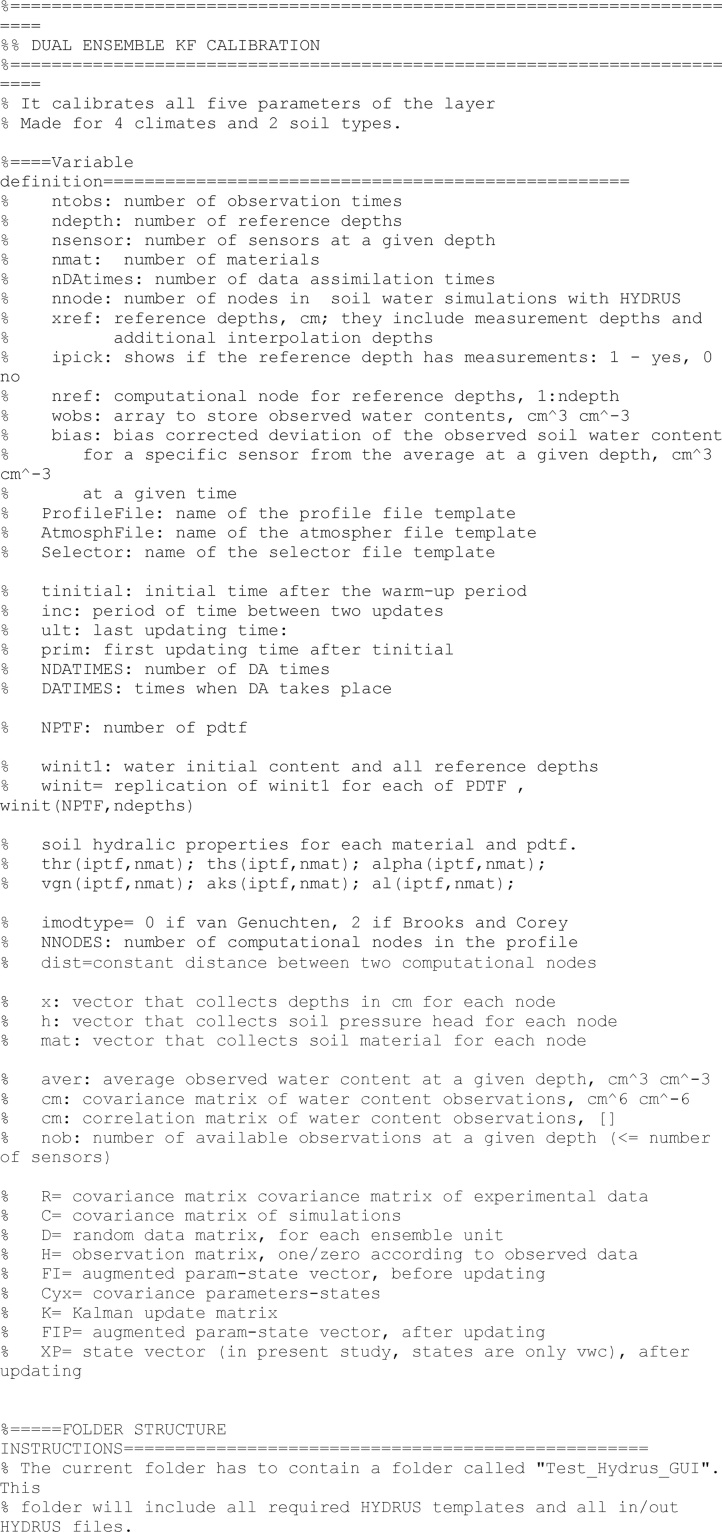

First lines clean all previous results and identify the folder of the input information.

**Figure:**
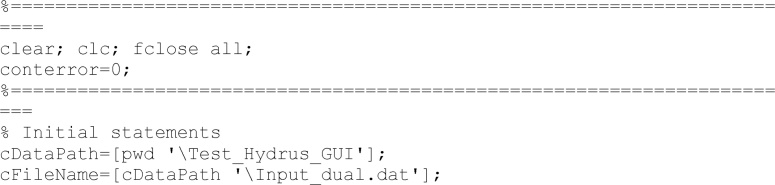

Following lines read the ‘InputSHP.dat’ file. This file contains the soil hydraulic information for the correct values, the initial values for the searching process, boundaries of existence domain for soil properties, etc.

**Figure:**
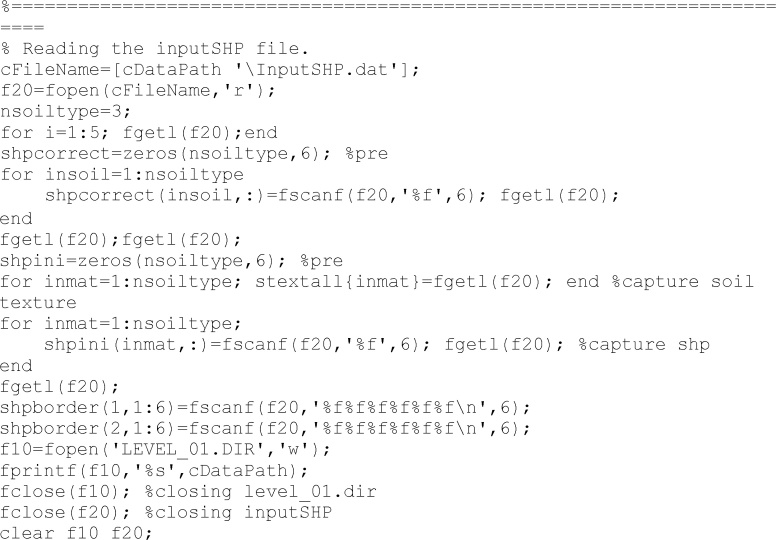

Next, time information is introduced. *tinitial* informed when the warm-up period finished and when the updating process begins. *inc* collects the time interval in days between different updatings. Time between *tinitial* and the first updating is collected in *prim* variable. Finally, *ult* collects the last day.

**Figure:**
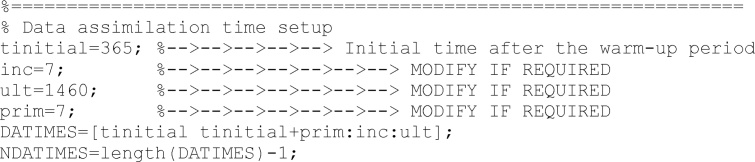

In the next step, geometrical information: Number of nodes, location of observation points, and others can be modified here.

**Figure:**
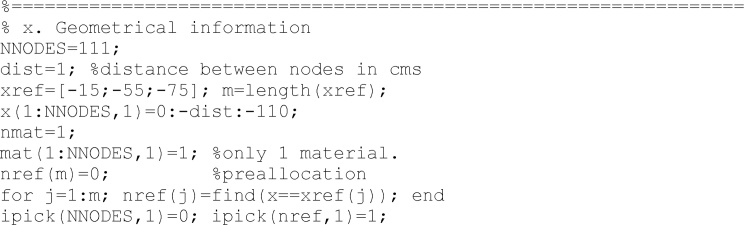

As abovementioned, the code is prepared to run different climates and soil types. Here the climate-soil loop starts

There are four different climate alternatives. Users may create new climate files following the same structure.

**Figure:**
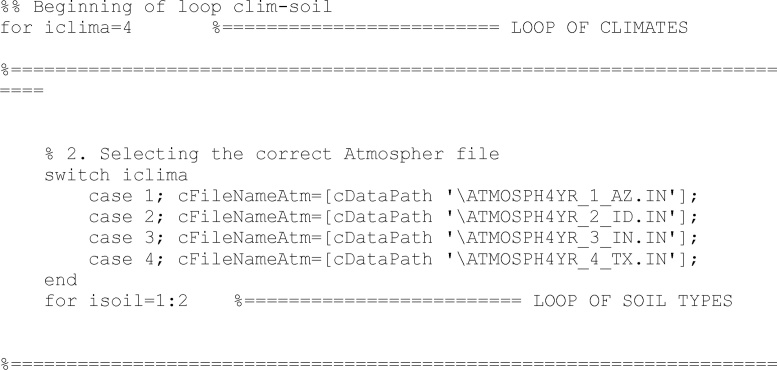

In the following lines, the code reads the observation data according to the selected climate and soil type. In Valdes-Abellan et al. [3] is exposed a method to create synthetic data.

**Figure:**
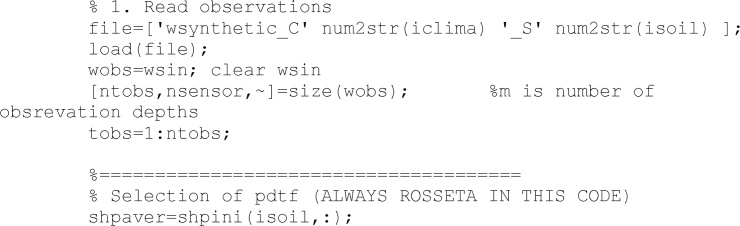

The ensemble of models is created in the following lines. Different alternatives can be chosen by changing the *generation* variable value. The first option uses the covariance matrix show in Faulkhner et al. [4]; the second uses a diagonal covariance matrix (i.e., only standard deviation are considered but not covariance between different parameters).

Additionally, users can choose the number of units in *nunit* variable. The code let the user to decide what soil parameters are going to be upated. *elecshp* is a logical variable: 1 means that the parameter will be considered during the updating process, and 0 means the opposite. *elecshp* length is five, according to *θ*r, *θ*s, *n*, α and *K*s.

**Figure:**
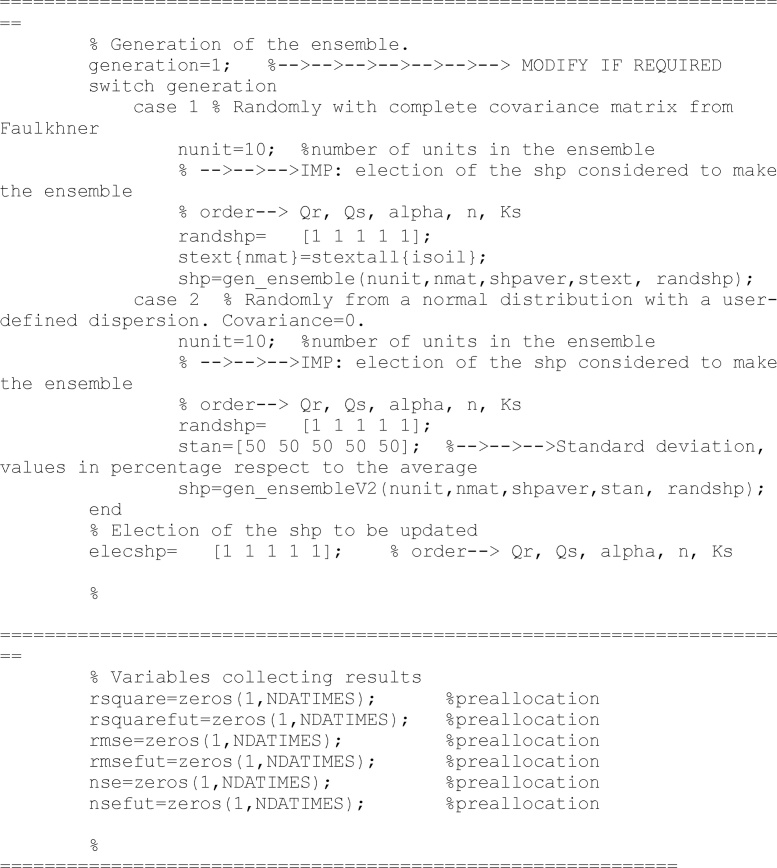

The previous lines create the variables where results will be saved. At this time they are equal to zero.

In the following the updating process for a specific soil-climate case begins from the initial time, after the warm-up period, to the end of the updating process

**Figure:**
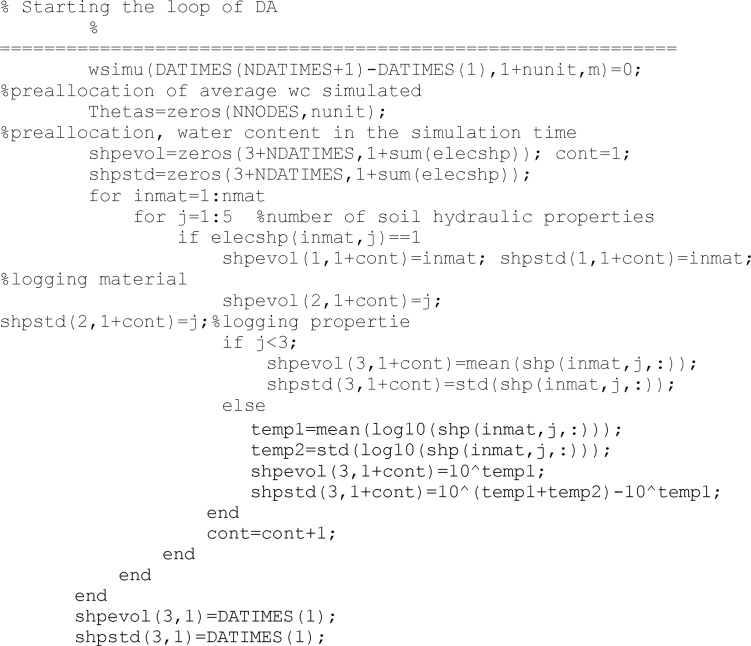

The Hydrus software is used to solve the Richards-equation-based soil water flux. It requires three input files: ATMOSPH.IN, PROFILE.DAT and SELECTOR.IN. The following code lines are devoted to create those files for each unit of the ensemble for the period ranging from the previous to the next updating time.

The PROFILE.DAT file requires the definition of the initial condition for the complete profile. Criterion to get this is to translate volumetric water content into soil pressure head according to the specific soil hydraulic properties in the observation depths (i.e., those depths were there were data) and to interpolate linearly soil pressure head between observation depths. This is made in the function called *h* = *W_TO_H_Bv3.* Other options were considered but finally were discarded.

**Figure:**
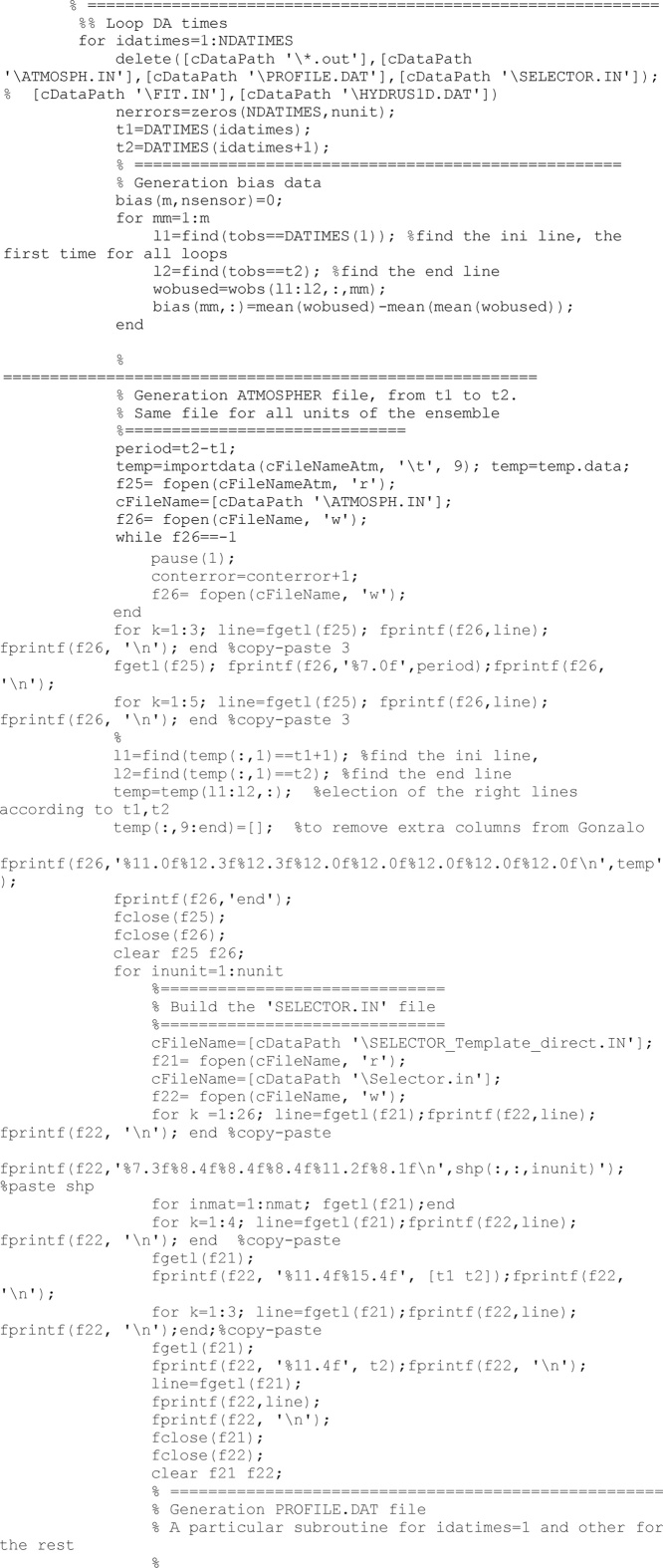

**Figure:**
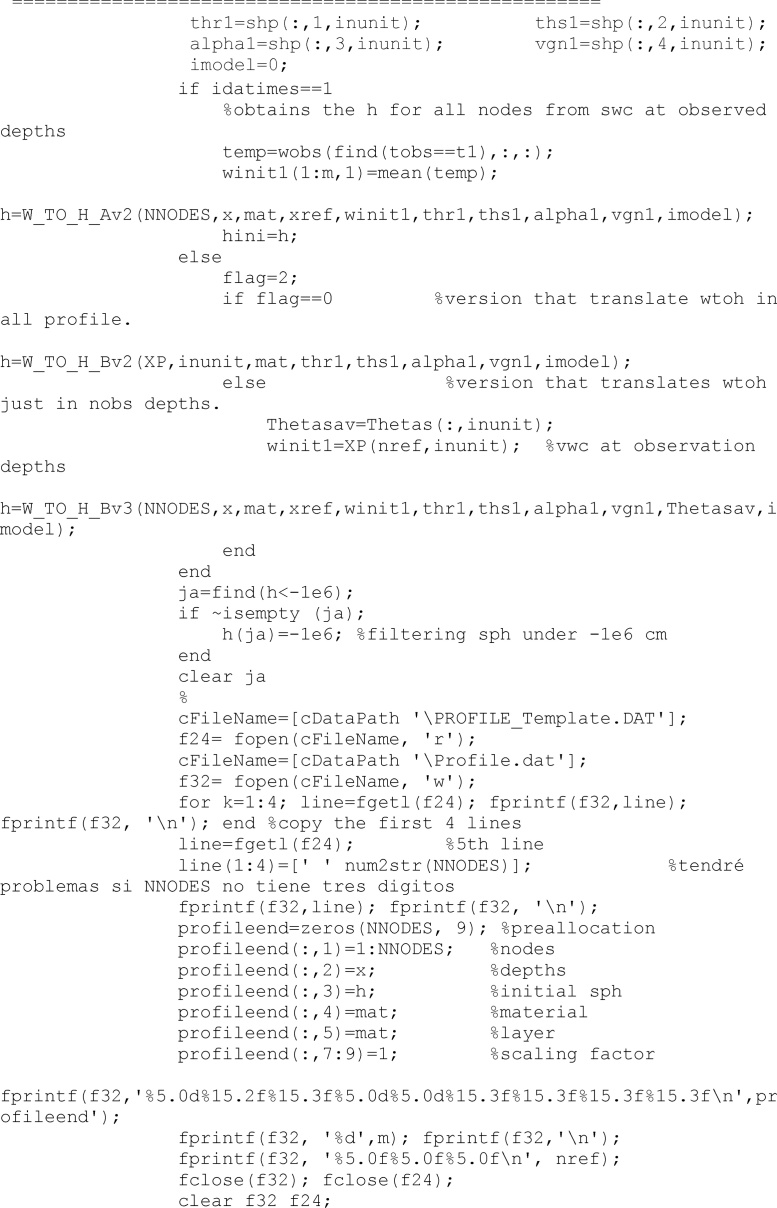

With all required files, HYDRUS is finalled called. In the present code, simulations requiring more than 6 s to finished computations were considered uncorrect and discarded. To interrupt a HYDRUS running, a system function called taskkill was used.

**Figure:**
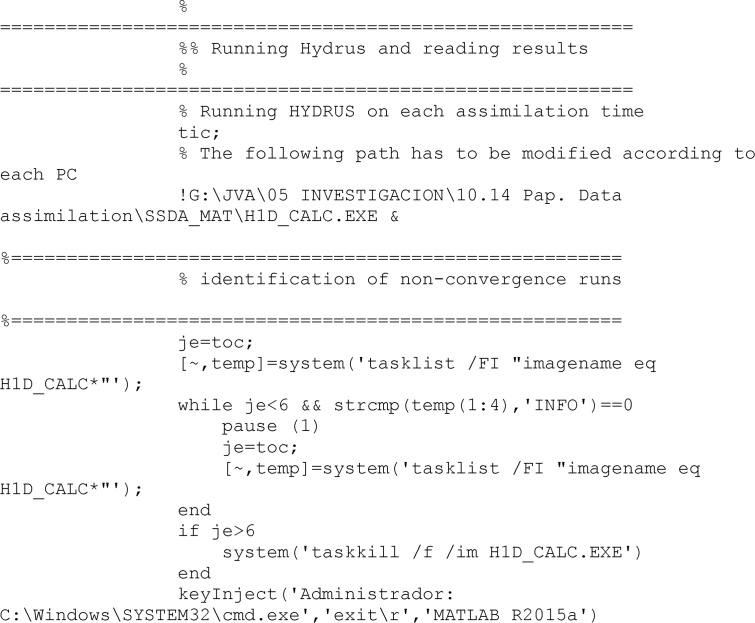

After computations finish, results from this run are read. This run implies a specific unit of the ensemble, a specific climate, a specific soil and a specific time.

**Figure:**
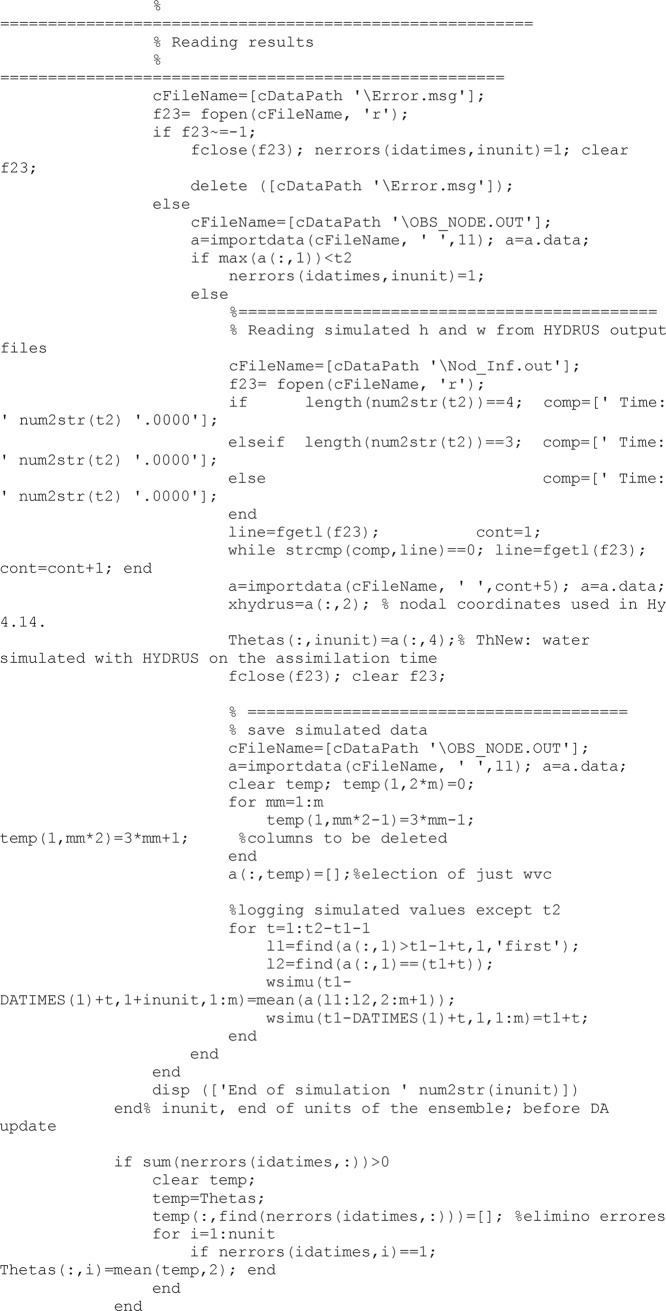

Once all units of the ensemble have been run and their results collected, the updating process can be undertaken. First, all required matrices are obtained (*R, X, C, D, H, Cyx*) and second an updated augmented vector of states and parameters (*FIP*) is obtained

**Figure:**
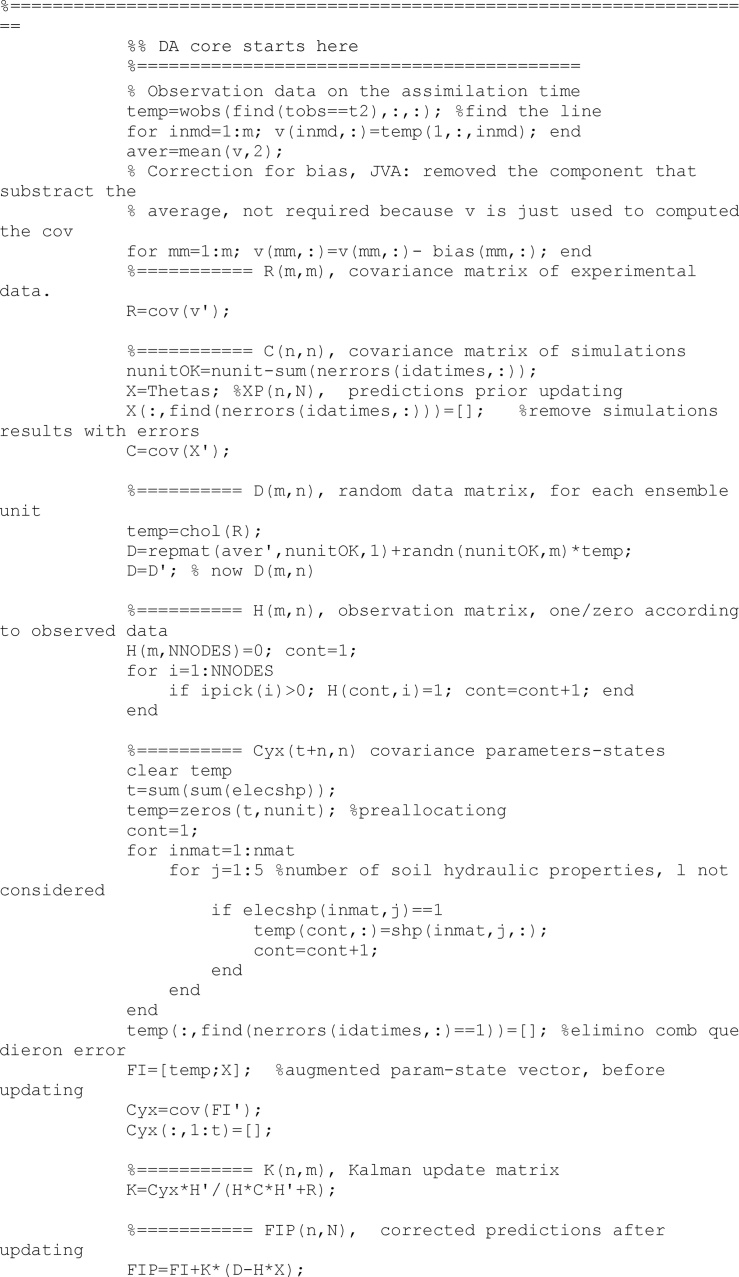

When the updated vector is obtained, averages values from the successful runs are assigned to the runs which reported an error and therefore they had no results.

**Figure:**
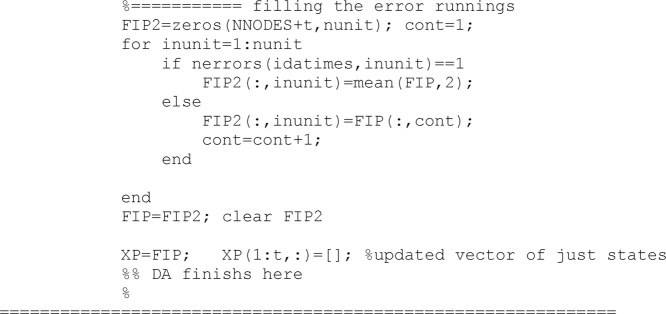

If updated soil parameters falls out of logical boundaries during the updating process (e.g., residual water content below zero), then they are moved to the closest border of a logical domain. Border values are included in the ‘InputSHP.dat’ file and have been read in the first stages.

**Figure:**
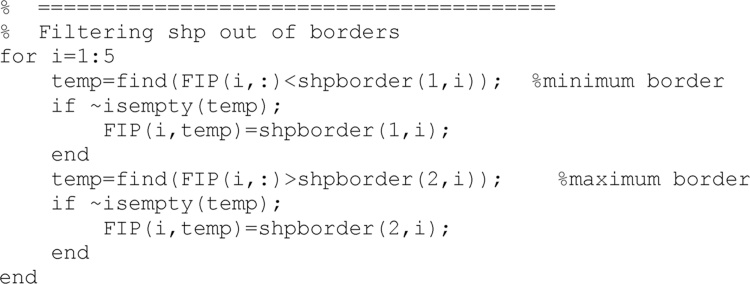

After the previous filter, results from soil hydraulic parameters updating are saved

**Figure:**
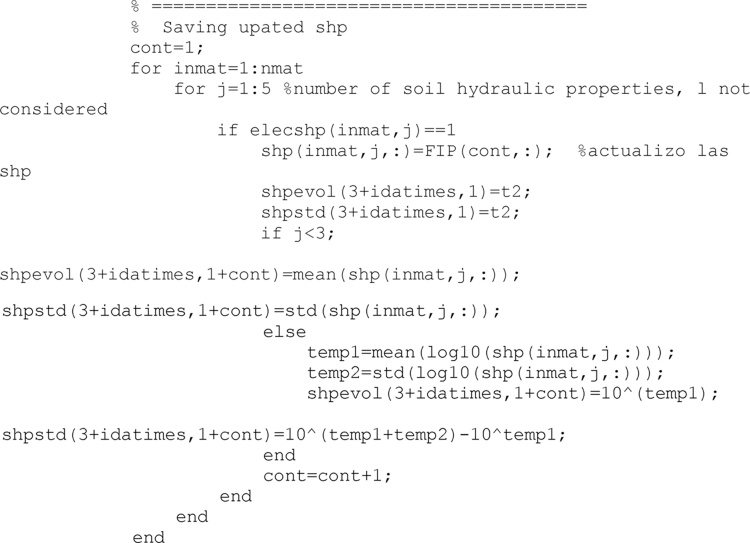

Soil states (volumetric water content and soil pressure head) are also corrected to avoid illogical values, similarly to the process undertaken with soil hydraulic properties.

**Figure:**
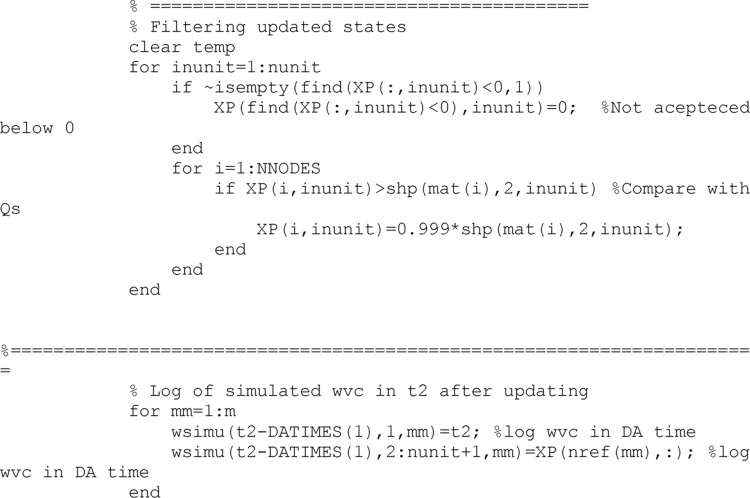

After the updating process has finished completely, and results have been saved, a direct run is developed with the last updated set of soil properties to obtain the model performance. The direct model is run from the beginning to the time of the last updating.

As all Hydrus runs, before to run the model, all required files have to be built. After the model has run

**Figure:**
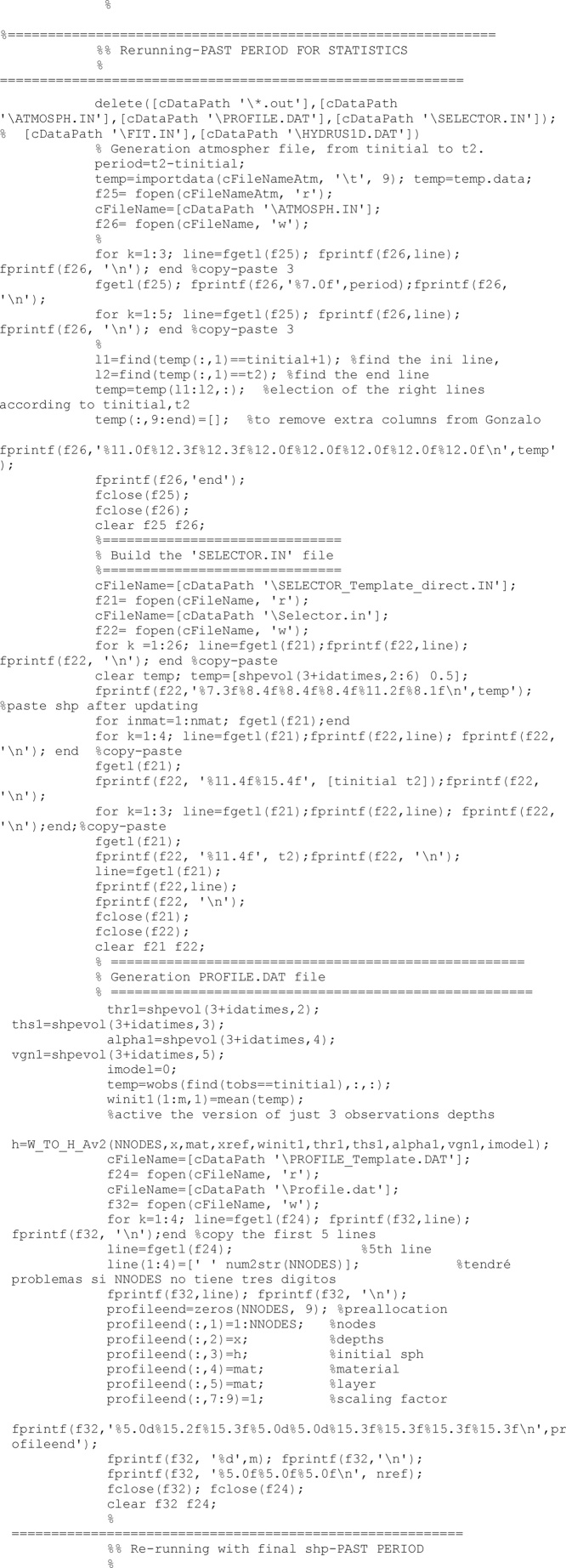

**Figure:**
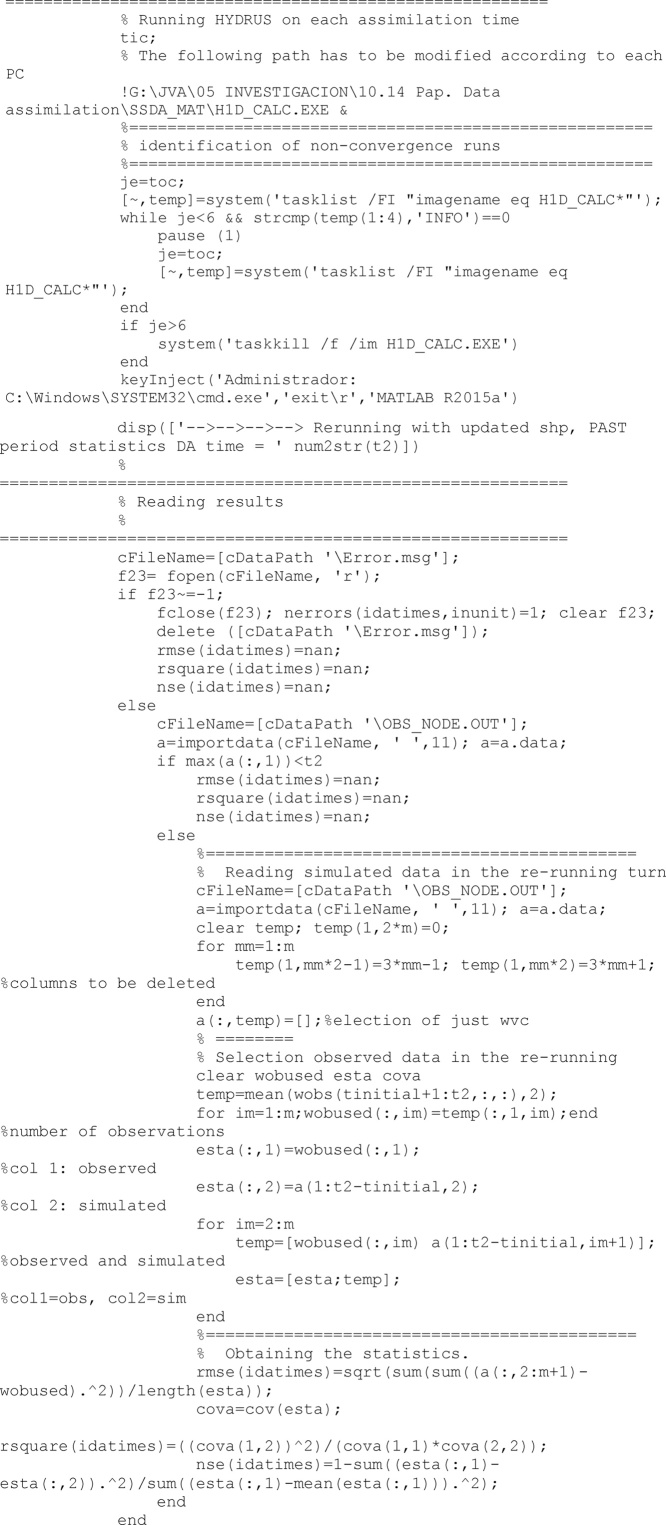

Similarly to the direct run for the past period, another direct run is accomplished with the last updated value of soil properties to obtain the model performance in case of future predictions. As abovementioned, first all required files are made, then Hydrus software is call. Finally the root mean square error, RMSE, the coefficient of determination, R^2^, and the Nash-Sutcliffe efficiency index, NSE [5], statistics are computed.

**Figure:**
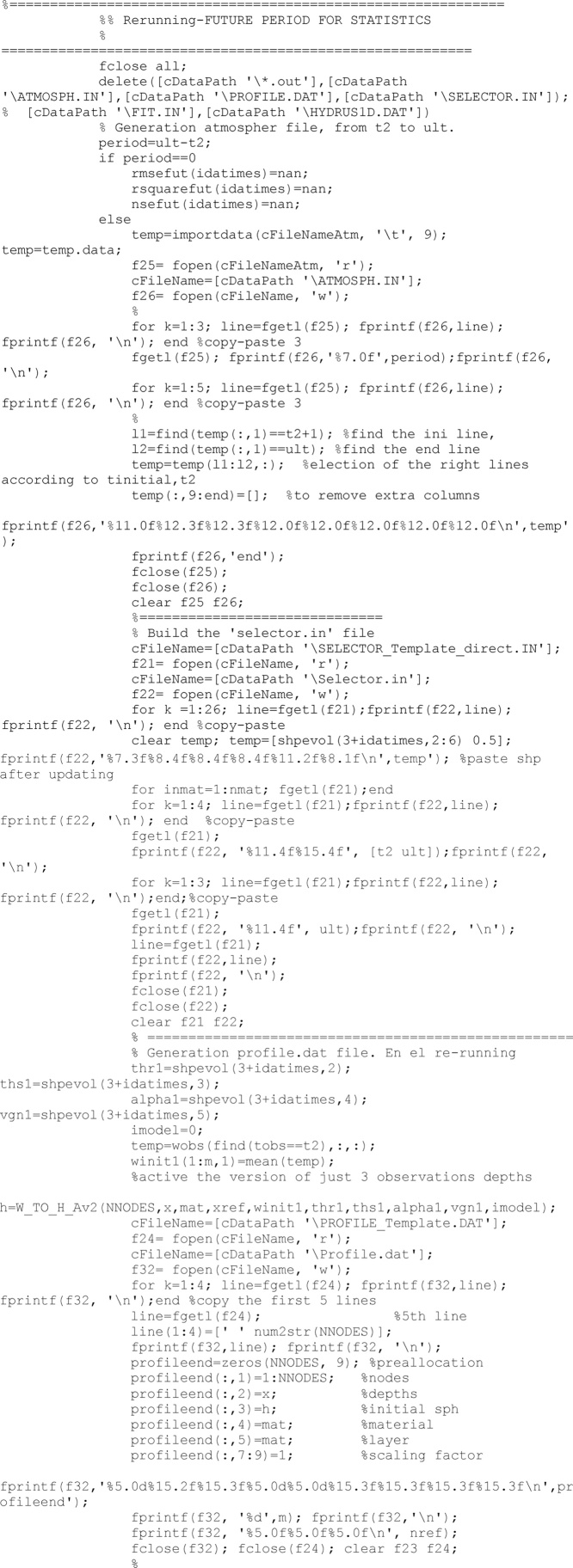

**Figure:**
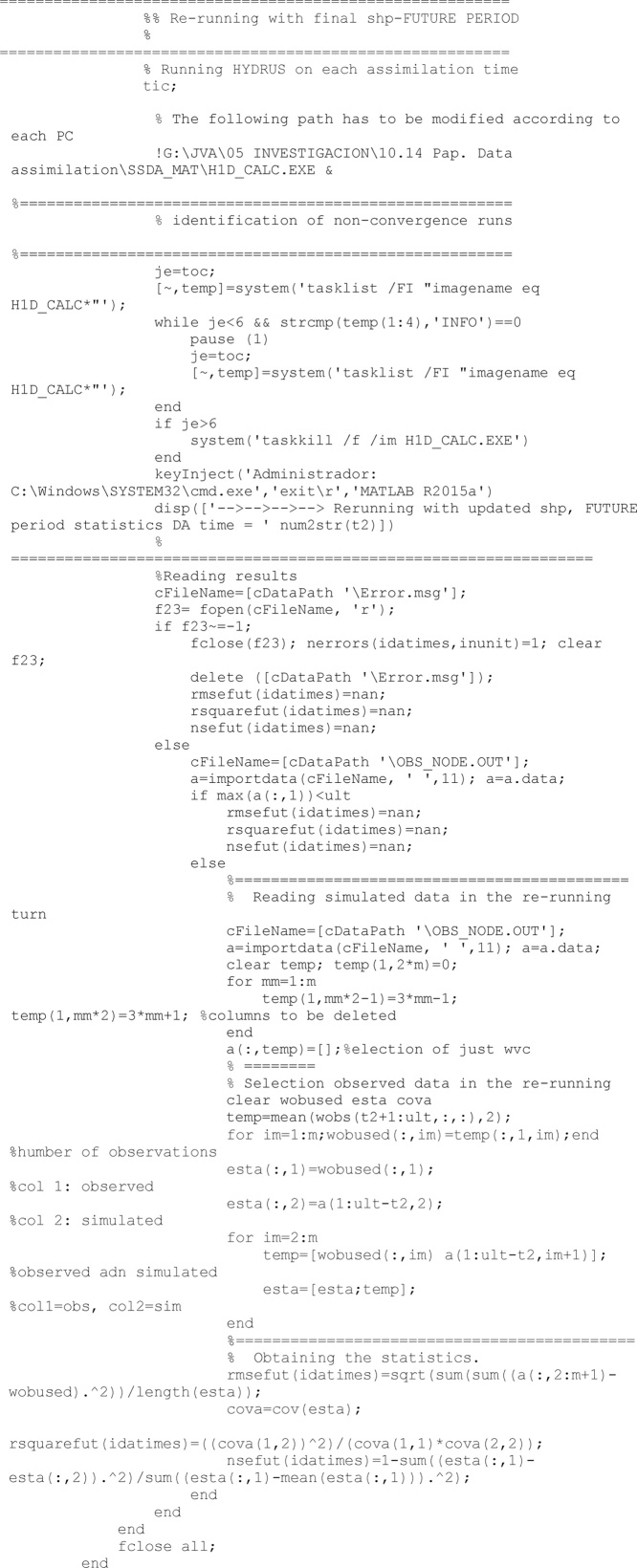

Last stages of the code are devote to save all results.

**Figure:**